# Disclosure of Parental HIV Status to Children: Experiences of Adults Receiving Antiretroviral Treatment at an Urban Clinic in Kampala, Uganda

**DOI:** 10.1155/2017/3458684

**Published:** 2017-10-25

**Authors:** Charles Peter Osingada, Monica Okuga, Rose Chalo Nabirye, Nelson Kaulukusi Sewankambo, Damalie Nakanjako

**Affiliations:** ^1^Makerere University College of Health Sciences, School of Health Sciences, Department of Nursing, P.O. Box 7072, Kampala, Uganda; ^2^Makerere University School of Public Health, Department of Health Policy Planning and Management, P.O. Box 7072, Kampala, Uganda; ^3^Department of Internal Medicine, School of Medicine, Makerere College of Health Sciences, P.O. Box 7072, Kampala, Uganda

## Abstract

Limited data are available on the experiences of parental HIV disclosure to children in Uganda. We conducted a qualitative study comprising sixteen in-depth interviews and four focus group discussions with parents receiving highly active antiretroviral therapy. Analysis was done using Atlas.ti qualitative research software. Back-and-forth triangulation was done between transcripts of the in-depth interviews and focus group discussions, and themes and subthemes were developed. Barriers to parents' disclosure included perceptions that children are too young to understand what HIV infection means and fears of secondary disclosure by the children. Immediate outcomes of disclosure included children getting scared and crying, although such instances often gave way to more enduring positive experiences for the parents, such as support in adherence to medical care, help in household chores, and a decrease in financial demands from the children. Country-specific interventions are needed to improve the process of parental HIV disclosure to children and this should encompass preparation on how to deal with the immediate psychological challenges associated with the parent's disclosure.

## 1. Introduction

HIV and AIDS continue to be a major public health challenge worldwide more so in sub-Saharan Africa where HIV-related illness is a leading cause of morbidity and mortality. Global estimates indicated that, by the end of 2014, 36.9 million people were living with HIV infection, and of these 25.8 million were in sub-Saharan Africa [1]. Even though there has been a global decline in new HIV infections and associated deaths, the number of clients enrolled for antiretroviral therapy has steadily grown to reach at least 15.8 million [1]. According to a national survey of 2011, about 7.3% of individuals aged 15–49 years are infected with HIV [2]; the number of new infections reportedly dropped from 128068 in 2009 to about 99000 in 2014, while the estimated number of annual HIV-related deaths declined from 52799 in 2009 to 32890 in 2015 [3]. A decrease in HIV-related deaths [4] coupled with an increase in the number of people accessing antiretroviral treatment has given birth to the phenomenon of social normalization of HIV and AIDS [5] which according to Roy and colleagues depends on both disclosure of HIV status to a person's social networks and willingness and ability of the network to support the emotional and material needs of the person living with HIV [6]. In work conducted in Uganda and Tanzania, high adherence resulted from social and material support from an individual's social networks [7].

HIV status disclosure has been the subject of numerous studies on the African continent, particularly in view of increasing incidence of HIV transmission to sexual partners in several countries [8–13]. Also, following publication of the World Health Organization (WHO) guidelines on HIV counselling for children up to 12 years of age [14], there has been renewed interest in encouraging parents and caregivers to disclose their HIV status to children. A parent's disclosure of their HIV-positive status can occur at different levels. It can be limited to sharing of health information between the parent and child which then happens to result in the child knowing the parent's serostatus. Disclosure can also be planned by the parent and shared either directly or indirectly: it can take the form of full disclosure when the terms HIV and AIDS are used in sharing the health information, resulting in the child knowing the parent's HIV-positive status; or it can take the form of partial disclosure when the specific terms HIV or AIDS are not used by the parent, yet the parent's actions or conversation results in the child becoming aware of the parent's HIV-positive status.

Available literature indicates that HIV-positive individuals struggle with what, when, and how to disclose their HIV-positive status [15–17]. Nondisclosure has been justified by parents based on the premise of protecting children from the negative social and psychological consequences caused by disclosure [16, 18–20]. Nevertheless, nondisclosure has been associated with negative consequences for both the parent and child [21, 22]. In many developing countries, there is a growing demand for culturally appropriate interventions intended to increase parents' readiness to disclose their HIV status to their children [15]. Interventions to encourage parental HIV disclosure to children have been tested in the United States [21, 23] and China [24]. However, in sub-Saharan Africa, with the exception of South Africa [25–27], such interventions are uncommon. Moreover, the implementation of these kinds of interventions should be grounded on a good understanding of the local context regarding potential parental HIV disclosure. In Uganda, limited data are available on the barriers, approaches, motivators, and consequences of parental disclosure of HIV status to children. This scarcity of information provided the impetus for this study.

## 2. Methods

### 2.1. Study Design and Setting

A cross-sectional, descriptive, qualitative study was conducted among HIV-positive clients in care and treatment at the Infectious Diseases Institute at Makerere University's College of Health Sciences, Kampala, Uganda. The Infectious Diseases Institute is a Ugandan not-for-profit organization, established in 2002, whose mission is to strengthen HIV-related care, training, and research in Africa. By 2014 the Infectious Diseases Institute was providing care and treatment services to over 110000 people living with HIV in urban and rural settings in Uganda, which amounts to about 13% of the national effort [18].

### 2.2. Participant Recruitment and Data Collection

The study participants comprised adult parents who were HIV-positive, who either had or had not disclosed their HIV serostatus to their child/children, and who had a biological child aged at least 5 years or older. The age-5 lower limit was chosen based on a recommendation by the World Health Organization [14] that children of school age should be told of a caregiver's HIV-positive status. No upper limit cut-off for a child's age was stated because in African settings parents tend to perceive children as a child irrespective of their child's age. The sample was purposively determined to include both clients who had disclosed their HIV status and those who had not. Potential participants were identified by the research assistants at the clinic; to avoid disrupting the smooth running of the clinic, willing clients were given appointments to return later for an in-depth interview (IDI) and/or focus group discussion (FGD). The selection involved a process of screening all potential participants during the time when they were waiting for review of their individual treatment. Each client who met the inclusion criteria was then given an appointment for when to return for the purposes of participating in the study. Sampling was also guided by the principle of data saturation—that is, data collection was terminated when no new information appeared to be elicited from the IDIs and FGDs.

Data were first collected from participants who returned for an IDI. Each parent was taken through the process of consent, and those who provided written informed consent were then individually interviewed by one of four trained research assistants. Each interview lasted between thirty and forty-five minutes and all the interviews were conducted in Luganda (the local language of central Uganda). To ensure privacy and confidentiality, each interview was conducted in a quiet room usually used for counselling clinic clients. Each of the participants in the study, in either an IDI or FGD, was paid the equivalent of US $10 to compensate for travel and time. A total of 16 IDIs were carried out with HIV-positive parents who had or had not disclosed their status to their child/children. Of these, nine parents had disclosed their HIV status (i.e., shared health information with a child, resulting in the child knowing the parent's HIV-positive status) and seven had not. In addition, four FGDs, each with 8 or 9 participants, were carried out: two groups with men and two groups with women. We chose to conduct separate FGDs for men and women for cultural reasons, as in the Ugandan context anecdotal evidence suggests that women tend to express their opinions less freely in the presence of men. We conducted one FGD for men and one for women who had disclosed their HIV status, and likewise there was one group for men and another for women who had not disclosed their HIV status. A total of 35 clients participated in the four FGDs. The IDIs and FGDs were conducted using guides developed by the investigators after reviewing the literature on parental HIV disclosure. Different guides were developed for research with parents who had disclosed their HIV status and with those who had not. All the guides were translated into Luganda by an expert from Makerere University's Department of Linguistics, English-Language Studies and Communication. The translated guides were pretested among HIV-positive parents attending the Old Mulago HID and AIDS Clinic in the general outpatient department, which was not part of the study site so as to avoid bias that would result from patients who participated in the pretest being included in the study. Adjustments were made to the flow of questions and probes before the data collection began.

### 2.3. Data Management

Audio-recordings of the IDIs and FGDs were transcribed and translated and then analysed by an expert with a social science background, using Atlas.ti qualitative data management software. The transcripts were assigned a name that did not contain any personal identifying information, to maintain anonymity. A code book was developed after reading through a random sample of five transcripts (2 FGDs and 3 IDIs) to ascertain the emerging themes. The transcripts were then entered into Atlas.ti. Next, the codebook from the emerging issues that had been transformed into different codes was entered into Atlas.ti. Each of the transcripts was then thoroughly read and any portion of text identified that related to a particular code was highlighted and attached to the respective code. Query reports for each of the codes were produced containing short summaries of texts. Each of these short summaries was read several times and analysed manually using manifest content analysis to identify emerging patterns. Back-and-forth triangulation was done between transcripts of the FGDs and IDIs before a decision was made to group those sources together under the themes and subthemes. These themes reflected underlying meanings in the text. In presenting the results here, we quote extractions from the texts, representing the themes, to support our findings.

## 3. Results

### 3.1. Sociodemographics of the Participants

A total of 51 parents on ART participated in the study. Sixteen clients participated in IDIs: 7 males and 9 females; their ages ranged from 28 to 59 years. Thirty-five individuals participated in FGDs: 19 males and 16 females; their ages ranged from 24 to 67 years. Religious affiliations and disclosure status of each participant are shown in Table 1.

### 3.2. Parents' Experiences regarding HIV Disclosure

Experiences concerning parental HIV disclosure fell into four categories: barriers/hindrances, motivators, approaches, and consequences. Themes that emerged from the data in each category are summarised in Table 2.

### 3.3. Barriers to Parental HIV Disclosure

Table 1 summarises four themes that emerged from the data relating to barriers or hindrances to parents' disclosure: viewing disclosure as a burden, perceived immaturity of the children, strength of the relationship between the parent and child, and anticipated unfavourable level of support from the child.

#### 3.3.1. Viewing Disclosure as an Unnecessary Burden

Some parents who had not disclosed their HIV status viewed sharing their serostatus as an unnecessary burden that was not worth the risk of going through it. They feared that their child/children might ask about their own HIV status, that they would probably tell the other partner/parent about their HIV test result, and that this would exert pressure on the parent to explain how the disease came into the family. One male participant in a FGD said the following:

Those children can put pressure on me…first of all, I have not taken them for testing, so I do not know whether they are [HIV] negative or positive. I have also feared to do this; I may take them to test and one or two are found [HIV] positive, and the woman will ask “how did it come?” And we might get misunderstandings in the home, so I kept quiet and I have peace…as you know we have responsibilities in some organizations and even in the central government, so it gave me peace for keeping my secret. (participant in men's FGD)

#### 3.3.2. Children Perceived as Too Young to Understand

Some parents chose not to disclose their HIV status because they perceived their child as too young to understand the meaning and consequences of HIV infection, and some also believed a child would not be able to keep the parent's HIV status confidential. One woman explained the following:

I take drugs with my husband, and [the children] donot see it, we keep it in the bedroom. Even if they go there they have not yet known it, they have never seen it anywhere. They will only see the tin, they will not know whether it is for headache or stomach pains. I will tell them when they grow up, but now they are very young, even if you tell them they will not take it seriously. (IDI with female)

#### 3.3.3. Poor Relationship between Parent and Child

Parents, but particularly fathers, often chose not to disclose their HIV status to their child because they felt the relationship between them was not good enough to facilitate disclosure. Some parents reported lacking the personal confidence to disclose their HIV status, and in other instances they felt their child was not interested in their health. Other reasons included parents viewing their adult children as being interested only in sharing the parent's property and less so in personal concerns. There were also cases where the children were regarded as being close to only one parent, which made the other uncomfortable about disclosure. This is demonstrated in explanations provided by two men:

Only that the other three I do not always get information about them, that is why I do not tell them some things; if I want I just pay their fees and they go to school, but they do not know some information about me…they are all spoiled and they now think like her [their mother]; they do not expect me to tell them anything good, they only respect what their mother tells them. So I have left them like that. (IDI with male)

“Another thing, I have many children and they are not from the same mother; so I think that if I tell, this one will think that maybe it is this mother who did this [brought the infection into the family], but there is not much problem. (IDI with male)

#### 3.3.4. Anticipated Unfavourable Level of Support from a Child

Some parents felt self-reliant because they were in otherwise good health and able to support themselves and thus did not feel the need or benefit of disclosure to their children. One man explained the following:

I do not have any serious problem…until when I reach my last day, so that gives me confidence that I do not need my children to know…so I saw that if I am capable I do not need to disclose…what I did, I got a house boy who helps me in everything…. I can be able to do everything on my own; I do not need any support, and whenever I need help I just run here. (participant in men's FGD)

### 3.4. Motivation of Disclosure

A parent's motivation to disclose their HIV status was captured in four themes (Table 3). Overall, men were more likely to be motivated to disclose their HIV status to their child so as to clarify doubts about their HIV-positive status, whereas females were more likely to disclose their HIV status as a result of seeing it as an obligation to their child.

#### 3.4.1. Parental Obligation of Disclosure

Some parents had disclosed their HIV status clearly because they felt it was their obligation and responsibility to their children. One mother remarked the following:

What forced me to disclose to them, when I produced children, I never produced a boy, I produced four children and they are all girls. One of them died and I remained with three, so it pained me a lot that if I do not tell these children…they are like my sisters, so I said these children should grow knowing my status, they do not have any brother in life, I am now their sister, so let me tell them. (IDI with female)

#### 3.4.2. Clarification of Doubts about the Parent's HIV Status

Some parents explained that their disclosure had arisen after they noticed suspicion among their children, who might have noticed “suspicious medicines” in the home or that the parent was attending monthly hospital appointments:

One time I was there and I thought I would die and these children would not know how I died or what killed me; and as you know when you die you cannot fail to get someone among ten people who will feel sorry, so I first told the boy; and I told him that I am very sick, and this disease is so serious that it does not cure, and he told me there is no disease that cannot cure; I told him that don't joke for me, I am sick, then I asked him that “have you ever seen people suffering from HIV/AIDS?” (IDI with male)

#### 3.4.3. Need to Protect the Child

Other parents had disclosed their HIV status out of a desire to protect adolescent children who were likely to engage in higher-risk sexual behaviours. One mother said the following:

So now she was grown, with breasts growing big, she started putting on short skirts, and that is when I called her and told her that I have HIV and it is the reason I take drugs every morning and evening. She got surprised and said that at school we learn that HIV is not cured…and I advised her not to go in for boys, and good enough at church they sign papers that they will not have sex until marriage, and for her she honours that commitment so much, and she always says “Mammy, I cannot sleep with boys, not until I put on a white gown like you did.” (mother of teenager, in FGD)

One father stated it this way:

My children are big, and they were seeing me taking my medicine, and they are also growing; so you have to show them that love is good but it also has tears; you have to show children that HIV kills and it is very painful and it is not fun to take medicine every day. (father of teenager, in FGD)

#### 3.4.4. Disclosure Based on Parent's Feeling of Improved Health

Several parents had been motivated to disclose their HIV status based on noticing an improvement in their health and thus felt their disclosure would less likely scare the child based on this timing:

And my daughter was worried and asking herself what I was suffering from, because I used to fall sick all the time during that time, but I could not tell her. I only used to tell her that they are still investigating, but they had already told me that I was HIV-positive. When I was admitted and I was badly off [very sick], I decided not to disclose at that moment, [instead] I told them when I came back from the hospital. (IDI with female)

### 3.5. Parents' Approaches to Disclosure

The process of parental disclosure elicited two main themes (Table 4): as a result of circumstances and preplanning by the parent as either direct or indirect disclosure.

#### 3.5.1. Circumstantial Disclosure

Some parents started the process of disclosing their HIV-positive status to their children following circumstantial prompts, such as when a child asked about the parent's HIV status or wanted to know their own status, or following death of a family member. One participant said the following:

The child was also born [HIV] positive, so the child was started on septrin [cotrimoxazole] and when the child grew up she asked her mother that “but mummy, for how long will I take this medicine?” So the mum responded that “We shall take it until God will decide for us.” Then the child asked me that “Daddy, I always see mummy taking medicine and she also gives me medicine, what is it for?” For me, I am open, so I told her A-B-C-D [and I] disclosed. (participant in men's FGD)

#### 3.5.2. Planned Conversation

For some parents the process of disclosure was planned well in advance and it entailed gathering all the children together for making a direct disclosure in a family meeting:

So I called them together at the same time in the same room and I told them that take care, “you protect yourselves, you are young and you are girls. I, your mother, I had not told you but I am HIV-positive, if you did not know.” (IDI with female)

#### 3.5.3. Leaving Medicines in the Open

Some parents initiated the process of disclosure by deliberately leaving their antiretroviral medicines out for children to see, without ever verbally disclosing their HIV-positive status. For example,

I did not tell them abruptly, like you are there and you want to counsel your children and you tell them that I am like this, I have gone to the hospital and they tested my blood and I was found HIV-positive. But I used to leave my drugs (medicine) on the table for them to see and know for themselves. (IDI with female)

### 3.6. Positive Outcomes of Disclosure

Positive outcomes of the parents' disclosure (Table 5) included themes wherein a child showed concern about the parent's health, parents received support from the children, children improved their behaviour, and the parent felt psychological relief after disclosure.

#### 3.6.1. Children Showing Concern about a Parent's Health

In some cases, once a parent had disclosed their HIV-positive status, a child started reminding them to take their medicines and to keep hospital appointments. One father said the following:

And when time comes they tell me that “Father, your medicine?” And sometimes I take like a week when I am away from home, but they will ask you that “Daddy, how many days will you spend there?” I tell them that may be one week, and they will pack medicines for two weeks, and they will tell you that “I have put for two weeks because you might reach there and anything can happen.” (IDI with male)

#### 3.6.2. Parents Receiving Emotional or Practical Support from Children after Disclosure

Some children had responded to their parent's disclosure by offering emotional, material, financial, or even food support to the parent. Their support also included help with household chores:

Since the children got to know about my status they provided a lot of care because they know their mum is sick; they do most of the work at home, especially the boy does not want me to go and fetch water and he fetches it himself…so that is it…they help me a lot because they do all the work. (IDI with female)

#### 3.6.3. Improved Behaviour of Children

There were cases when children were described as making excessive financial demands before the parent had disclosed their HIV status, yet after disclosure the parent reported a positive change in a child's behaviour and this lessened the parent's stress. One woman said the following:

So they understood and they felt concerned, and they started to give me support and they reduced on the demands they were putting on me; those who used to ask money for special hire to take them to school started taking a taxi to go to school. (IDI with female)

#### 3.6.4. Parents' Psychological Relief

Some parents reported a sense of relief after disclosure to their children. One mother said the following:

Those other ones were also hurt, but one of the children was more hurt, she showed that it was too much for her…. In fact I did not feel okay after telling them, but after some days I felt I had offloaded a very big luggage from my head, because it had haunted me always and I always wanted to tell them, but there was no way I would tell them because I thought it would hurt them. (IDI with female)

### 3.7. Negative Consequences of Disclosure

Two themes commonly emerged in both the FGDs and IDIs concerning negative consequences from a parent's disclosure: emotional breakdown of either the child or parent and children getting scared by the information.

#### 3.7.1. Emotional Breakdown

For most of the parents an immediate negative outcome of disclosure had been children and, in some cases, even the parent crying afterwards as, for instance, the following:

Then I asked him that “have you even heard about HIV?” He told me, HIV? Daddy, HIV kills, they tell us at school that HIV kills,” and he told me HIV is AIDS, he was trying to explain. Then I told him that I take that medicine because I was tested and found that I was HIV-positive, the HIV that kills, but I take that medicine so that I can prolong my life. Then he broke down into tears, he cried; so I told him that “This is the reason I did not want to tell you when you are still young, but that is it.” (IDI with father of teenage boy)

They tried to cry a bit saying, “our daddy died and our mother's life is also in suspense,” but I consoled them and told them that “Let us leave everything in God's hands; you pray for me, support me, so that I can take my medicines, because the moment I miss taking it, it is bad.” (IDI with female)

#### 3.7.2. Children Getting Scared

In other cases when parents had disclosed their HIV status, the children became very scared and stressed, and this also worried the parents themselves:

These children [6 and 12 years] were already in school, and they could teach them about HIV at school, but they were in great fear that every day mummy has been having HIV, which means she is going to die. They were stressed, and as a parent you would see that what you have told them did them bad, they were so much affected. (IDI with female)

My children [ages 6 and 12 years] realized it themselves and I never sat them down, they had already seen their dad suffering, and the elder boy asked me that “Mummy, in which health facility do you seek care?” And I told him that they had directed me to the infectious diseases clinic…the young one got scared and I told him to be firm. (participant in women's FGD)

## 4. Discussion

As supported by our findings, parents' HIV status disclosure to children is still a challenging issue characterised by numerous obstacles, varied approaches, and mixed outcomes. We found it interesting that parents who perceived themselves as self-reliant and in otherwise good health did not consider it essential to disclose their HIV-positive status to their children. In addition, however, we observed many of the usual other hurdles to disclosure, as previously described in the literature, such as parents' perceptions that children are too young to understand the health information and that they will be subsequently unable to keep it secret [16, 18, 28–30], fear of having to explain how a parent became infected [31], and perceptions of a poor relationship between the parent and child [29].

In this study, parents' motivation for disclosure sometimes clearly related to their improved health status following an episode of severe illness. To our knowledge, this finding has not been reported by other studies in Uganda. Parents had also often disclosed their HIV status out of a sense of responsibility and obligation to their children. Similar findings have been reported by studies conducted in the United States [32] and China [33]. Importantly, this finding provides an opportunity for healthcare professionals to build on parents' inner strength and desire to protect, beyond just making the simple recommendation that parents should disclose their HIV status to their children. Thus, future interventions could aim to bolster parents' propensity to discharge their perceived responsibility in this regard. Moreover, parents who are provided with basic information and skills about disclosure are likely to be encouraged and better equipped to disclose their HIV status to their children, and this can include the benefits of improved family communication and cohesion [34, 35].

Some parents in this study cited the need to protect adolescent children from engaging in higher-risk sexual behaviour as a strong consideration for their disclosure. This finding supports the results of studies conducted in China [33], Burkina Faso [29], and the United States [35]. One implication is that the promotion of HIV status disclosure, particularly in the context of family, may pay significant dividends in regard to HIV prevention. This is so because parents who discuss with children measures to prevent HIV infection are more likely to be trusted and believed than other adults because of their experience and position in the family. This supports the need for approaches that enhance parental self-efficacy in HIV disclosure. Whereas in this study disclosure was often portrayed as a protective action, the literature is rife with examples where nondisclosure has been justified by parents based on the same premise of protecting children from the negative social and psychological consequences caused by disclosure [16, 18–20]. Nevertheless, while nondisclosure is sometimes intended to protect children, it has been associated with negative consequences for both the parent and child [21, 22]. Additional research, especially in resource limited countries with a high burden of HIV, is merited to demonstrate whether the benefits of parental disclosure far outweigh the negative outcomes.

In this study, the process of disclosure for some parents was hastened by circumstances, such as children asking parents how long they (child/children) were going to be on a medication. For other parents, disclosure was a preplanned process that involved gathering their children together for a family discussion which culminated in the parent sharing his or her HIV-positive status. Similarly, Demattero and colleagues (2002) reported that disclosure was sometimes planned in the context of gathering for a special meal or favourite family activity. The same authors stated that disclosures had also occurred in the context of a family argument or after a parent had been drinking. The experiences of parents in this study and others reveal that there is a dearth of practical and generalizable approaches to help parents in making decisions regarding the process of disclosure. Future work in this area, especially in Uganda and similar African contexts, should focus on developing contextually appropriate interventions to encourage parents' HIV status disclosure to children.

In this study, the outcomes of parental HIV disclosure were mixed, as has been similarly demonstrated in other studies. Positive outcomes noted by parents after disclosure included children reminding the parent about taking their medication, packing medicines for the parent when they had to travel, helping more with household chores, and improving their behaviour, including reducing the financial demands the child put on the parent; comparable findings were reported by Kennedy et al. (2010) and Tiendrebeogo et al. [29]. Conversely, negative consequences of disclosure tended to be the immediate emotional burden, as many of the parents reported that a child had reacted by crying or getting scared. Perhaps it is primarily for fear of these emotional reactions that many parents avoid disclosure to children [36]. Yet, despite the negative consequences associated with parental HIV disclosure, it is suggested that parents' nondisclosure to children might actually lead to increased psychosocial vulnerability [22].

## 5. Study Limitations

The findings of this qualitative study cannot be generalized to all HIV-positive parents in Uganda. Furthermore, when interviews are used as a method of data collection, there is a tendency of participants to distort their responses to present a favourable opinion, which leads to response bias. This was minimized by emphasizing honesty throughout the data-collection period. Finally, it would have been interesting to document the consequences of parental HIV disclosure from the perspectives children, but this was outside the scope of this study. Despite these limitations, the study presents important findings that can help shape the direction of interventions to promote parental HIV disclosure in Uganda. Approaches to facilitate parental HIV disclosure in Uganda and other highly affected resource limited settings are especially needed following the recently released World Health Organization policy guidelines requiring all individuals who test HIV-positive to commence ART [37]. These policy guidelines have resulted in development of country-specific “test and treat” guidelines thus transforming HIV into a chronic infection.

## 6. Conclusions

Parental HIV disclosure remains a complex phenomenon hampered by psychological barriers and uncertain family dynamics. In situations where disclosure occurs, the outcomes may be mixed for both the parent and child. However, HIV-treatment programmes in sub-Saharan Africa require culturally suitable interventions that facilitate the process of disclosure. Particularly, interventions are needed to better prepare parents and children to deal especially with the immediate psychological problems associated with parental HIV status disclosure. In the meantime we recommend that parents and children should receive counselling before and after disclosure to deal with psychological challenges associated with disclosure.

## Figures and Tables

**Table 1:** Sociodemographic characteristics of study participants.

Number	Gender	Age	Focus group discussion (FGD)/in-depth interview (IDI)	Region	Disclosure status
1	Female	32	FGD	Protestant	Not disclosed
2	Female	41	FGD	Protestant	Not disclosed
3	Female	26	FGD	Muslim	Not disclosed
4	Female	49	FGD	Protestant	Not disclosed
5	Female	29	FGD	Catholic	Not disclosed
6	Female	24	FGD	Protestant	Not disclosed
7	Male	54	FGD	Protestant	Disclosed
8	Female	67	FGD	Protestant	Disclosed
9	Female	26	FGD	Catholic	Disclosed
10	Female	46	FGD	Protestant	Disclosed
11	Female	45	FGD	Protestant	Disclosed
12	Female	60	FGD	Protestant	Disclosed
13	Female	41	FGD	Protestant	Disclosed
14	Female	38	FGD	Catholic	Disclosed
15	Male	42	FGD	Protestant	Disclosed
16	Male	54	FGD	Catholic	Disclosed
17	Male	51	FGD	Catholic	Disclosed
18	Male	37	FGD	Protestant	Disclosed
19	Male	40	FGD	Muslim	Disclosed
20	Male	58	FGD	Muslim	Disclosed
21	Male	39	FGD	Protestant	Not disclosed
22	Male	39	FGD	Catholic	Disclosed
23	Male	31	FGD	Protestant	Not disclosed
24	Male	61	FGD	Protestant	Not disclosed
25	Male	42	FGD	Catholic	Not disclosed
26	Male	40	FGD	Protestant	Not disclosed
27	Male	50	FGD	Protestant	Not disclosed
28	Male	45	FGD	Protestant	Not disclosed
29	Male	40	FGD	Protestant	Not disclosed
30	Female	30	FGD	Catholic	Not disclosed
31	Male	46	FGD	Protestant	Disclosed
32	Male	52	FGD	Protestant	Not disclosed
33	Male	58	FGD	Protestant	Disclosed
34	Female	45	FGD	Pentecostal	Disclosed
35	Female	33	FGD	Catholic	Not disclosed
36	Male	37	IDI	Protestant	Disclosed
37	Female	50	IDI	Protestant	Disclosed
38	Female	45	IDI	Muslim	Not disclosed
39	Male	28	IDI	Catholic	Not disclosed
40	Female	44	IDI	Muslim	Not disclosed
41	Female	45	IDI	Pentecostal	Disclosed
42	Male	46	IDI	Catholic	Disclosed
43	Male	59	IDI	Protestant	Not disclosed
44	Female	56	IDI	Pentecostal	Disclosed
45	Female	44	IDI	Protestant	Not disclosed
46	Female	48	IDI	Muslim	Not disclosed
47	Male	33	IDI	Protestant	Disclosed
48	Female	35	IDI	Catholic	Disclosed
49	Male	43	IDI	Muslim	Not disclosed
50	Male	49	IDI	Protestant	Disclosed
51	Female	48	IDI	Catholic	Disclosed

**Table 2:** Barriers/hindrances to disclosure as presented by parents who did not disclose HIV serostatus to their children.

Themes	Subthemes
Viewing disclosure as a risk or burden	(i) Parent being in poor health and therefore concerned more about their health than disclosure. (ii) Parents perceived the children as already knowing their (parent's) HIV status. (iii) Parents feared that the children may ask them about their own (child's) HIV status. (iv) Parents feared their children's assumption that they (parents) are about to die. (v) Parents preferring to have “peace of mind” and hence avoid disclosure to children.

Immaturity of children	(i) Children were viewed by the parent as being unable to understand the meaning and consequences of being HIV-positive. (ii) Parents observed that the child might not keep the parent's HIV-positive status confidential.

Relationship between parent and child	(i) Relationship between parent and the children was viewed as not good enough to facilitate disclosure. (ii) Parents reported not being personally confident enough to disclose their HIV status. (iii) Children did not seem interested and passionate enough about the health of their parents. (iv) Parents viewed their children as being interested only in sharing the parent's property. (v) Children being closer to one of the parents, making the other uncomfortable to disclose their HIV status under such circumstances.

Anticipated support from children	(i) Parent viewed children as not being able to support them emotionally or practically. (ii) Parent still felt in good health and saw no need to be supported by the children.

**Table 3:** Motivators to disclosure, as presented by parents who disclosed their HIV status to their children.

Theme	Subthemes
Obligation of disclosure	(i) Parent wanting to clarify their HIV-positive status to the children. (ii) Desire to prepare the children so that death of the parent would not come as a shock. (iii) Parents' desire to confirm what the children had earlier suspected that the parent might be HIV-positive.

Clarification or “clearing the air” about the parent's HIV status	(i) Desire to clarify the “suspicious medicines” (antiretroviral drugs) that the children had already seen the parent taking. (ii) Clarifying to the children about monthly hospital appointments.

Need to protect children	(i) Parents chose to disclose their HIV status as a way of warning them that higher-risk sexual behaviour could lead to contracting HIV. (ii) Desire to show children that HIV can “kill” or is “very painful” or that it is not pleasant to take medicines every day.

Improved health	(i) Parents disclosed their HIV status after noticing an improvement in their health.

**Table 4:** Some processes of disclosure, as presented by the patients that had disclosed their HIV status to their children.

Themes	Subthemes
Being prompted by events	(i) When the children asked about the parent's HIV status.
	(ii) When the children wanted to know their own HIV status.

Gathering children	(i) Parents planning well in advance and gathering the children together for the disclosure.

Offering clues	(i) Leaving the antiretroviral medicines for the children to see, without telling them that the parent is HIV-positive.

**Table 5:** Consequences of disclosure, as presented by parents that had disclosed their HIV status.

Themes	Subthemes
*(a) Positive consequences*	
Support and concern about health of the parent	(i) Reminding parents when the time for taking their drugs approached. (ii) Reminding their parents about the next hospital appointment. (iii) Financial assistance or helping with household chores.
Improved behaviour of children	(i) Change in the behaviour of the child to avoid stressing the parent.

*(b) Negative consequences (mainly short term)*	
Emotional breakdown	(i) Children responding to disclosure by crying. (ii) The parent themselves crying after disclosure. (iii) Stress for both parent and child.
Children getting scared	(i) Children responding by getting very scared.
